# Deep Ensemble Learning and Explainable AI for Multi-Class Classification of Earthstar Fungal Species

**DOI:** 10.3390/biology14101313

**Published:** 2025-09-23

**Authors:** Eda Kumru, Aras Fahrettin Korkmaz, Fatih Ekinci, Abdullah Aydoğan, Mehmet Serdar Güzel, Ilgaz Akata

**Affiliations:** 1 Graduate School of Natural and Applied Sciences, Ankara University, 06830 Ankara, Türkiye; ekumru@ankara.edu.tr; 2 Faculty of Health Sciences Nutrition, Dietetics Department, Şirinevler Campus, İstanbul Kültür University, 34191 Istanbul, Türkiye; a.korkmaz@iku.edu.tr; 3 Institute of Artificial Intelligence, Ankara University, 06100 Ankara, Türkiye; fatihekinci@ankara.edu.tr; 4 Department of Computer Engineering, Faculty of Engineering, Ankara University, 06830 Ankara, Türkiye; 21290200@ogrenci.ankara.edu.tr (A.A.); mguzel@ankara.edu.tr (M.S.G.); 5 Department of Biology, Faculty of Science, Ankara University, 06100 Ankara, Türkiye

**Keywords:** deep learning, explainable AI, fungal classification, ensemble models, morphological similarity, earthstar fungi

## Abstract

The current study presents a multi-class, image-based classification of eight morphologically similar macroscopic Earthstar fungal species (*Astraeus hygrometricus*, *Geastrum coronatum*, *G. elegans*, *G. fimbriatum*, *G. quadrifidum*, *G. rufescens*, *G. triplex*, and *Myriostoma coliforme*) using deep learning and explainable artificial intelligence (XAI) techniques. For the first time in the literature, these species are evaluated together, providing a highly challenging dataset due to significant visual overlap. Eight different convolutional neural network (CNN) and transformer-based architectures were employed, including EfficientNetV2-M, DenseNet121, MaxViT-S, DeiT, RegNetY-8GF, MobileNetV3, EfficientNet-B3, and MnasNet. The accuracy scores of these models ranged from 86.16% to 96.23%, with EfficientNet-B3 achieving the best individual performance. To enhance interpretability, Grad-CAM and Score-CAM methods were utilised to visualise the rationale behind each classification decision. A key novelty of this study is the design of two hybrid ensemble models: EfficientNet-B3 + DeiT and DenseNet121 + MaxViT-S. These ensembles further improved classification stability, reaching 93.71% and 93.08% accuracy, respectively. Based on metric-based evaluation, the EfficientNet-B3 + DeiT model delivered the most balanced performance, with 93.83% precision, 93.72% recall, 93.73% F1-score, 99.10% specificity, a log loss of 0.2292, and an MCC of 0.9282. Moreover, this modeling approach holds potential for monitoring symbiotic fungal species in agricultural ecosystems and supporting sustainable production strategies. This research contributes to the literature by introducing a novel framework that simultaneously emphasises classification accuracy and model interpretability in fungal taxonomy. The proposed method successfully classified morphologically similar puffball species with high accuracy, while explainable AI techniques revealed biologically meaningful insights. All evaluation metrics were computed exclusively on a 10% independent test set that was entirely separate from the training and validation phases. Future work will focus on expanding the dataset with samples from diverse ecological regions and testing the method under field conditions.

## Simple Summary

This study explores how artificial intelligence can help accurately identify and classify eight visually similar Earthstar mushroom species using photographs. For the first time, these mushrooms were analysed together as a group, which is challenging because their appearance often overlaps. The research tested eight advanced computer models called convolutional neural networks and transformers to see which could best tell the species apart. The most successful models reached over 96% accuracy. The study also combined two models into “ensemble” systems, which improved consistency and reliability in classification. To ensure the results are understandable, special techniques called Grad-CAM and Score-CAM were used to show which features in the images the computer focused on when making decisions. This approach makes artificial intelligence more transparent and trustworthy for scientists. Importantly, the findings could help monitor mushroom populations in nature and support sustainable farming practices by making fungal identification faster and more reliable. Overall, this work introduces a new and practical method for classifying mushrooms with both high accuracy and clear explanations for the results.

## 1. Introduction

Artificial intelligence-based image processing methods have gained increasing adoption in recent years, owing to their ability to deliver high accuracy, speed, and scalability in biological classification tasks [1,2]. In the field of mycology, this technological advancement provides a significant advantage, particularly when distinguishing morphologically similar species [3,4]. Earthstar fungi, such as *Geastrum*, *Myriostoma*, and *Astraeus*, are ectomycorrhizal species found globally, primarily in tropical and temperate regions. Their fruiting bodies lack a stipe and possess a three-layered peridium, with the outer layer forming a star shape as it opens. The inner layer houses the gleba, which releases spores through a bellows-like mechanism. These fungi primarily inhabit land but also thrive on decaying organic matter, including wood, dung, and leaf litter [5].

Within this context, classification studies of eight visually similar species with star-shaped external structures *Astraeus hygrometricus* (Pers.) Morgan, *Geastrum coronatum* Pers., *G. elegans* Vittad., *G. fimbriatum* Fr., *G. quadrifidum* Pers., *G. rufescens* Pers., *G. triplex* Jungh., and *Myriostoma coliforme* (Dicks.) Corda [5,6,7,8,9] provide a representative case for assessing the potential contributions of AI-based modelling to fungal taxonomy. Owing to their high visual similarity, these species are often misidentified by traditional methods, making them an ideal reference set for testing automated recognition systems [10,11].

The classification of Earthstar fungi species has long presented a taxonomic challenge, primarily due to the morphological features that fluctuate in response to environmental influences and the presence of highly similar visual patterns across species [10,12]. Variations observed within individuals of the same species, as well as overlapping traits among different taxa, complicate the classification process [13]. Subtle differences in peristome shape, surface texture, and colour often become indistinct in low-resolution or variably angled images [10,11,14]. These conditions necessitate expert interpretation during manual identification, which introduces subjectivity and potential errors. Therefore, robust deep learning algorithms and interpretable models that can visually justify their classification choices are required to address these challenges [15,16].

Eight deep learning architectures MobileNetV3, EfficientNetV2-M, RegNetY-8GF, DenseNet121, MaxViT-S, EfficientNet-B3, DeiT, and MnasNet cover a wide range of models that differ in depth, parameter count, and computational complexity [10,17,18,19]. These models include lightweight architectures optimised for efficiency as well as deeper, high-capacity networks designed for rich feature extraction [10,11]. To boost robustness and generalisation, training procedures typically use data augmentation strategies and class-balanced sampling schemes [20]. Beyond individual models, attention-based ensemble approaches such as EfficientNet-B3 + DeiT and DenseNet121 + MaxViT-S combine complementary feature representations from different architectures. Interpretability is tackled through explainable AI methods, specifically Grad-CAM and Score-CAM, which generate spatial attribution maps highlighting the salient regions that influence model predictions. This approach allows for a comprehensive evaluation of both predictive accuracy and the biological plausibility of decision mechanisms [10,11].

The present study contributes novel insights to the intersection of mycology and artificial intelligence through systematic model comparisons and analyses focused on explainability for fungal species that are challenging to identify morphologically. While the individual EfficientNet-B3 model achieved the highest classification performance across all metrics, the attention-based ensemble models outperformed the majority of single architectures, enhancing class separation and reducing error rates. By providing an open-access high-resolution dataset and reproducible model outputs, the study fosters a sustainable research environment. The findings also lay a robust foundation for broader applications beyond taxonomy, such as ecological monitoring, biodiversity documentation, and biotechnological discovery.

## 2. Materials and Methods

**Figure 1:**
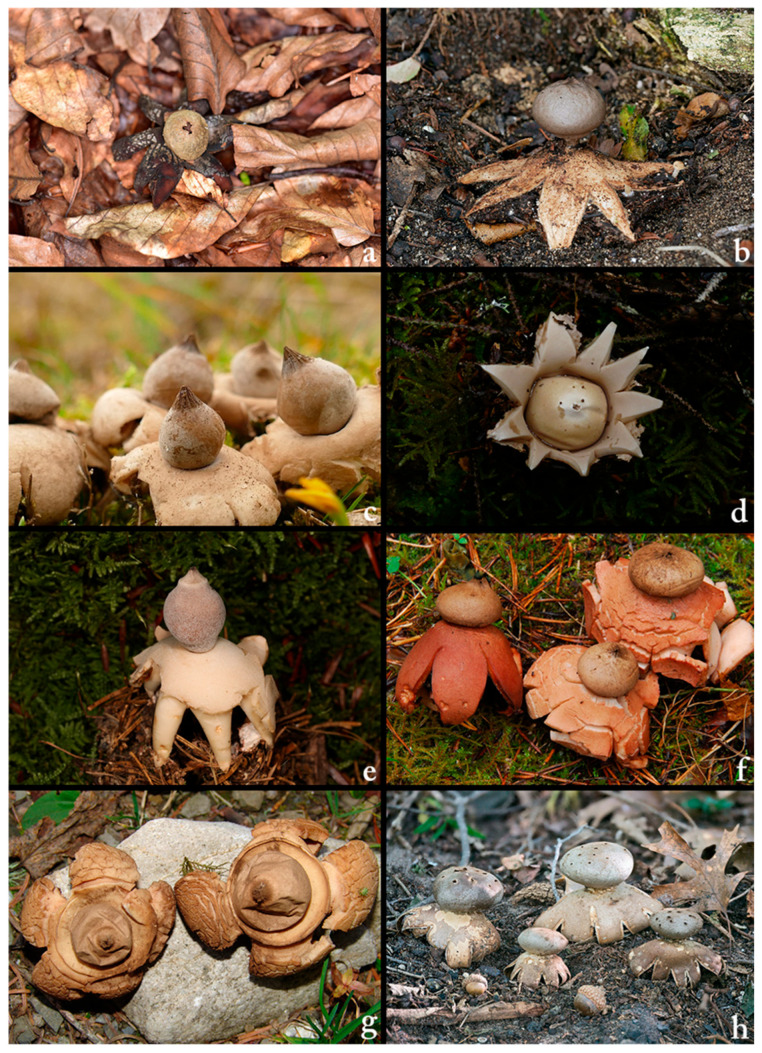
(**a**) *Astraeus hygrometricus*, (**b**) *Geastrum coronatum*, (**c**) *G. elegans*, (**d**) *G. fimbriatum*, (**e**) *G. quadrifidum*, (**f**) *G. rufescens*, (**g**) *G. triplex*, (**h**) *Myriostoma coliforme*.

The dataset utilised in this study comprises high-resolution images of eight different fungi species: *Astraeus hygrometricus*, *Geastrum coronatum*, *G. elegans*, *G. fimbriatum*, *G. quadrifidum*, *G. rufescens*, *G. triplex*, and *Myriostoma coliforme* (Figure 1).

A total of 1585 images were collected, with each class containing 200 images, except for *Geastrum coronatum*, which included 185 samples. Approximately 5% of the images were captured by the authors, while the remaining 95% were sourced from publicly available platforms such as the Global Biodiversity Information Facility [21]. All images were saved in JPEG format at a standard resolution of 300 dpi. Despite a slight imbalance among classes, the dataset was considered sufficiently balanced for multi-class classification tasks. An overview of the percentage of images obtained from each source, the training, validation, and testing image distributions for each class, and the continents where the photographs were taken is provided in Table 1.

**Figure 2:**
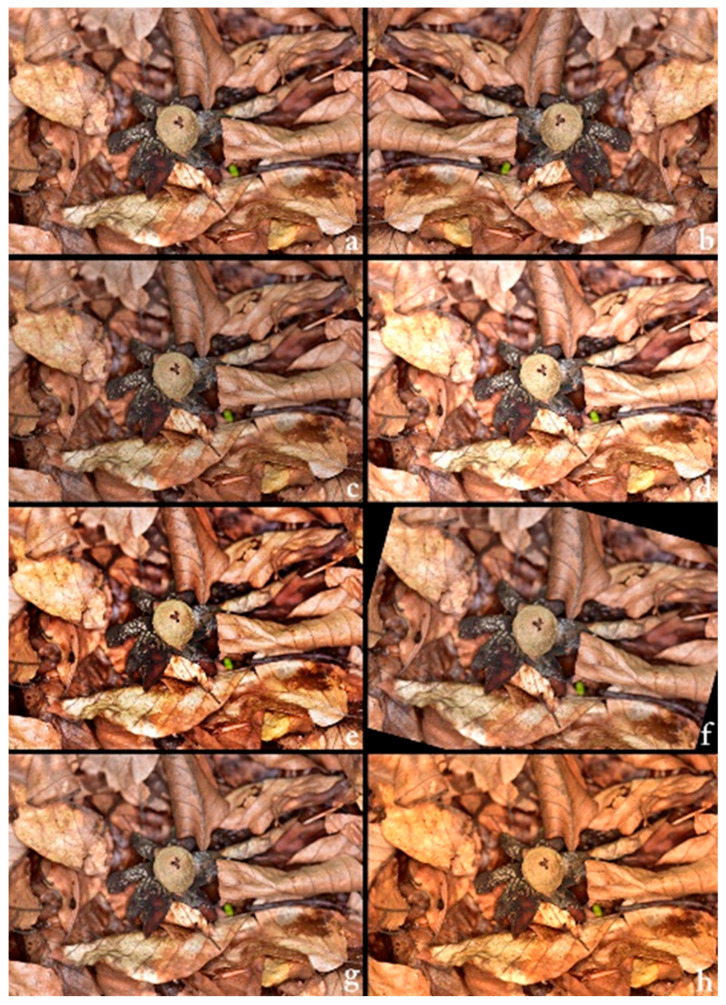
Example of various image augmentation techniques applied to a sample of *Astraeus hygrometricus*. (**a**) Original image, (**b**) horizontally flipped version, (**c**) brightness-reduced version (−25%), (**d**) brightness-enhanced version (+25%), (**e**) contrast-enhanced version (+60%), (**f**) rotated version (+15°), (**g**) blurred version (Gaussian radius = 4), (**h**) color-enhanced version (+80%). The figure illustrates how diverse augmentations are used to simulate varying real-world imaging conditions, enhancing the robustness and generalization ability of the deep learning model.

Before commencing model training, the dataset was randomly divided into three subsets: 80% for training, 10% for validation, and 10% for testing, as indicated in Table 1. This stratified split ensured consistent class representation across all stages of experimentation. To enhance generalisation and emulate real-world variability, four augmentation techniques were applied exclusively to the training set: horizontal flipping, random rotation within ±15 degrees, brightness adjustment (±25%), and centre cropping (90% of the central region, resized back to original dimensions). For each training image, a random combination of three out of the four methods was utilised. Each original image produced three additional augmented variants, resulting in a fourfold increase in training data volume (Figure 2). All images were normalised using the standard ImageNet preprocessing values: means of (0.485, 0.456, 0.406) and standard deviations of (0.229, 0.224, 0.225) for the RGB channels, respectively.

The study incorporated a diverse set of convolutional and transformer-based architectures to evaluate classification performance across various model families. MobileNetV3, known for its lightweight structure and speed-optimised design, is particularly suited for resource-constrained environments [22]. EfficientNetV2-M offers a balance between accuracy and training efficiency by leveraging progressive learning and fused MBConv layers [18,23]. RegNetY-8GF, a scalable architecture designed with a design space exploration approach, emphasises regularity and computational adaptability [24]. DenseNet121 stands out with its dense connectivity pattern, promoting efficient feature reuse [25] and mitigating the vanishing gradient problem. MaxViT-S, a hybrid vision transformer [26], combines convolutional and attention mechanisms to capture both local and global dependencies. EfficientNet-B3 utilises compound scaling to optimise depth, width, and resolution for improved performance [27]. DeiT (Data-efficient Image Transformer) introduces transformer efficiency into vision tasks without relying on large datasets or external distillation [28]. Lastly, MnasNet employs a mobile-friendly neural architecture search (NAS) strategy to strike a balance between latency and accuracy, making it suitable for edge applications [19].

Alongside these individual architectures, the study presented two ensemble models crafted to combine the complementary strengths of both convolutional and transformer-based designs. These ensembles aimed to improve predictive robustness and capture more comprehensive feature representations by merging outputs from different model families.

**Figure 3:**
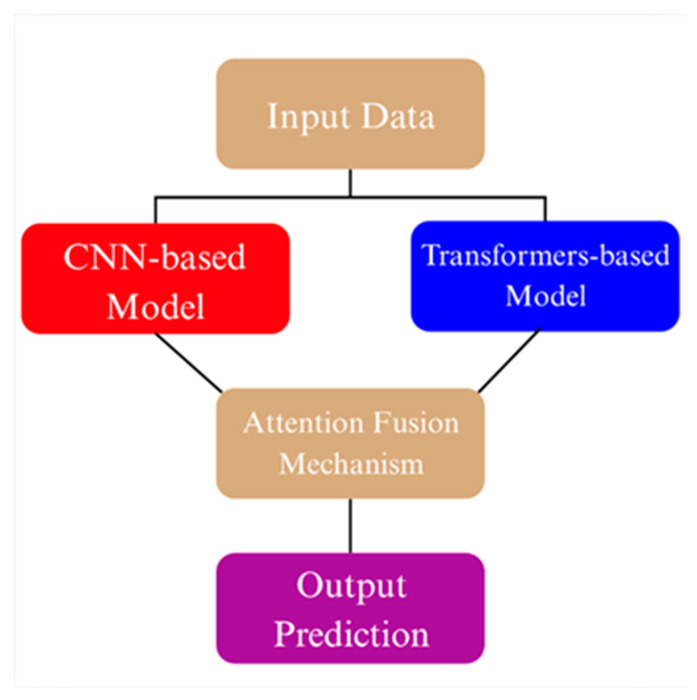
Conceptual illustration of the attention fusion mechanism used in ensemble models.

To effectively integrate the complementary representations of convolutional and transformer-based backbones, an ensemble fusion strategy based on learnable attention has been employed. In this architecture, features extracted from the two selected backbones EfficientNet-B3 and DeiT in the first ensemble, and DenseNet121 and MaxViT-S in the second are first linearly projected into a shared embedding space of 512 dimensions using separate fully connected layers. The projected embeddings are concatenated and passed through a lightweight attention module consisting of a single linear layer followed by a softmax activation, which computes adaptive weights indicating the relative importance of each backbone’s feature vector [29,30]. These weights are then used to produce a weighted sum of the two feature streams, resulting in a fused representation. This final fused vector is fed into a three-layer multilayer perceptron (MLP) classifier comprising two hidden layers with 256 and 128 units, respectively, each followed by ReLU activations, and a final softmax output layer corresponding to the number of classes [31,32]. The attention-guided fusion not only improves robustness by exploiting cross-architecture complementarity but also enhances interpretability by dynamically modulating the contribution of each model based on input characteristics (Figure 3) [33,34].

A diverse range of deep learning architectures was selected for this study to evaluate their performance in the multi-class classification of mushroom species [10,11]. These models include convolutional networks, vision transformers, and hybrid architectures, ranging from lightweight designs to more computationally intensive structures. The key architectural details of all selected models, including type, input resolution, parameter count, and attention mechanism, are summarised in Table 2.

All models were trained using stochastic gradient descent (SGD) with a momentum of 0.9 and an initial learning rate of 0.005. A two-stage training strategy was adopted, comprising 10 initial training epochs followed by 10 fine-tuning epochs with a reduced learning rate of 0.0005. The loss was computed using categorical cross-entropy, and weight decay regularization with a coefficient of 1 × 10^−4^ was applied to mitigate overfitting. To adjust the learning rate dynamically, a ReduceLROnPlateau scheduler was employed, featuring a period of two epochs and a reduction factor of 0.5. All models were initialised with pretrained ImageNet weights, and the backbone layers were fine-tuned rather than frozen. Early stopping was not applied during training to ensure full convergence across all models.

For a thorough assessment of model performance in multi-class classification, we used a suite of standard evaluation metrics. These included accuracy, precision, recall, F1-score, specificity, log loss, and the Matthews correlation coefficient (MCC) [35]. Accuracy gives an overall measure of correct predictions, while precision and recall evaluate the model’s ability to accurately identify positive cases. The F1-score, as the harmonic mean of precision and recall, balances these two aspects [10,11]. Specificity measures the proportion of true negatives correctly classified, which is particularly useful in imbalanced scenarios. Log loss assesses the confidence of probabilistic predictions, penalising incorrect but overconfident outputs. Lastly, the MCC provides a robust single-value summary that accounts for all four elements of the confusion matrix and is especially informative in imbalanced classification tasks [10,11].

(1) $$Accuracy=\frac{TP+TN}{TP+TN+FP+FN}$$

(2) $$Precision=\frac{TP}{TN+FP}$$

(3) $$Recall=\frac{TP}{TP+FN}$$

(4) $$F1-Score=\frac{2\times (Precision\times Recall)}{Precision+Recall}$$

(5) $$Specificity=\frac{TN}{TN+FP}$$

(6) $$Log\;Loss=-\frac{1}{N}\sum_{i=1}^{N}\sum_{j=1}^{M}y_{ij}\log\left(\hat{y}ij\right)$$

(7) $$MCC=\frac{\left(TP\times TN\right)-\left(FP\times FN\right)}{\sqrt{\left(TP+FP\right)\times \left(TP+FN\right)\times \left(TN+FP\right)\times \left(TN+FN\right)}}$$

In our dataset, TP corresponds to the number of correctly identified images of each mushroom species (Equations (1)–(3) and (7)), whereas TN (true negative) refers to the images that are correctly recognized as not belonging to a given species (Equations (1), (2), (5) and (7)). Conversely, FP (false positive) indicates misclassified negative cases (e.g., non-Geastrum images labelled as Geastrum) (Equations (1), (2), (5) and (7)), while FN (false negative) indicates the number of positive instances that were misclassified as negative (Equations (1), (3) and (7)). In Equation (6), the term yij is a binary indicator variable that equals 1 if sample i belongs to class j, and 0 otherwise. Here, N represents the total number of samples in the dataset, and M denotes the number of target classes in the multi-class classification task [10,11,35].

In addition to the classical evaluation metrics, several auxiliary metrics were recorded to capture the practical aspects of model deployment. The macro-averaged Area Under the Curve (AUC) was employed to assess class-wise separability across all categories [10,11,35]. The average inference time per image was measured to quantify computational efficiency. The average maximum predicted probability and entropy values provided insight into model confidence and uncertainty. Furthermore, memory usage and GPU utilisation percentages were tracked, and an energy efficiency score was calculated to assess the environmental footprint of each model [10,11,36].

Alongside the standard evaluation metrics, confusion matrices were computed for each model to provide a class-wise breakdown of prediction performance. These matrices visualise the distribution of correct and incorrect predictions across all eight mushroom categories, offering insight into systematic misclassification patterns that may not be evident in aggregate scores such as accuracy or F1-score [10,11].

Deep learning models, despite their remarkable performance across a diverse range of vision tasks, are frequently criticised for their lack of transparency. These models function as “black boxes,” delivering predictions without providing human-understandable reasoning. This opacity presents significant challenges, particularly in sensitive areas such as medical diagnosis or scientific research, where comprehending the rationale behind decisions is essential. To tackle this limitation, the field of Explainable Artificial Intelligence (XAI) has emerged. XAI encompasses a set of techniques designed to interpret and visualise the internal workings of machine learning models, aiming to enhance their outputs’ transparency, trustworthiness, and actionability. By shedding light on which aspects of an input most influenced the model’s prediction, XAI bridges the gap between high model accuracy and human interpretability [10,11].

Two prominent methods for explaining decisions, Grad-CAM and Score-CAM, were used to interpret how deep learning models make decisions. Grad-CAM uses gradients from backpropagation to highlight important areas in the final convolutional layers, while Score-CAM relies on changes in class scores caused by masking activations, providing smoother and more robust attribution maps. The use of these methods is particularly advantageous for fine-grained classification tasks such as morphologically similar fungal species, as they produce coherent and biologically interpretable visual explanations that preserve spatial context [10,37].

For transformer models such as DeiT, patch token embeddings were extracted from the final self-attention block and reshaped into a pseudo-spatial format by excluding the class token and arranging the patch tokens into 2D grids [38,39]. This reconfiguration facilitated the generation of Grad-CAM and Score-CAM maps that emulate the spatial interpretability of CNNs. Grad-CAM and Score-CAM highlight local regions of interest while also capturing broader spatial context, thereby supporting biologically interpretable model explanations [10,37].

In ensemble configurations, attribution was conducted both at the backbone level and on the fused representation. Each backbone (EfficientNet-B3, DeiT, DenseNet121, MaxViT-S) was analysed individually using both XAI methods, and the fused embeddings were interpreted directly to reveal the integrated attention learned by the fusion mechanism. The rationale for using Grad-CAM and Score-CAM lies in their capacity to produce class-discriminative, spatially coherent, and biologically interpretable heatmaps, offering clearer insights than perturbation-based methods such as LIME and SHAP [10,37].

## 3. Results

This section presents an in-depth assessment of the proposed deep learning models for classifying fungal species. The evaluation includes examining validation behaviour, test set performance, confusion matrix analysis, model calibration, computational efficiency, and explainability. Both quantitative metrics and qualitative visualisations are used to assess each aspect, with the goal of providing a comprehensive understanding of the models’ robustness, reliability, and suitability for practical deployment.

**Figure 4:**
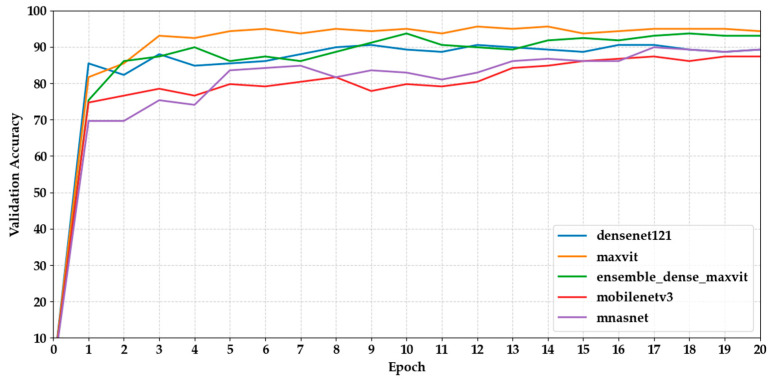
Shows the validation accuracy progression of the first five models over 20 epochs.

**Figure 5:**
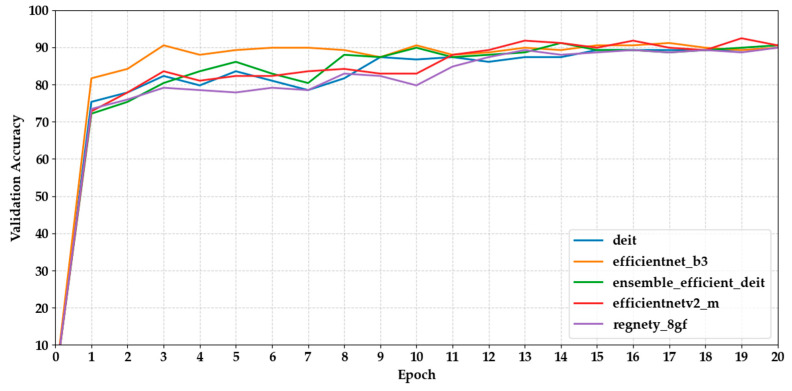
Shows the validation accuracy progression of the last five models over 20 epochs.

Figure 4 and Figure 5 show how the validation accuracy changes over 20 epochs for the first and last five models. Most models showed a steady increase, with the performance stabilising after about 10 epochs. Models based on transformers, such as Maxvit-S, and ensemble models like DenseNet121 + MaxViT-S made fast progress early on, reaching over 90% accuracy within the first 7 epochs. In contrast, lightweight CNNs like MobileNetV3 took longer to converge, needing more epochs to reach a plateau.

**Figure 6:**
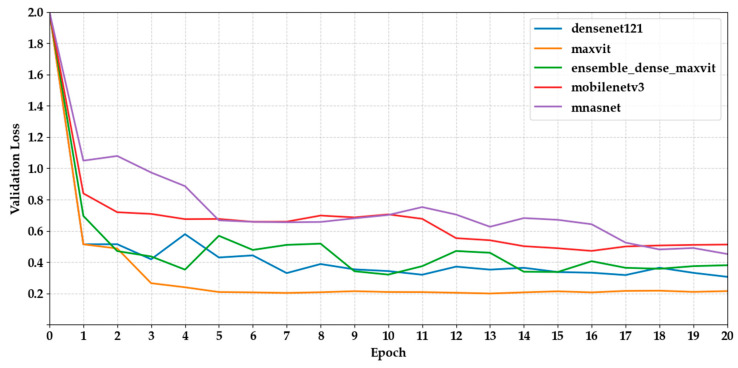
Depicts the validation loss curves of the first five models across epochs.

**Figure 7:**
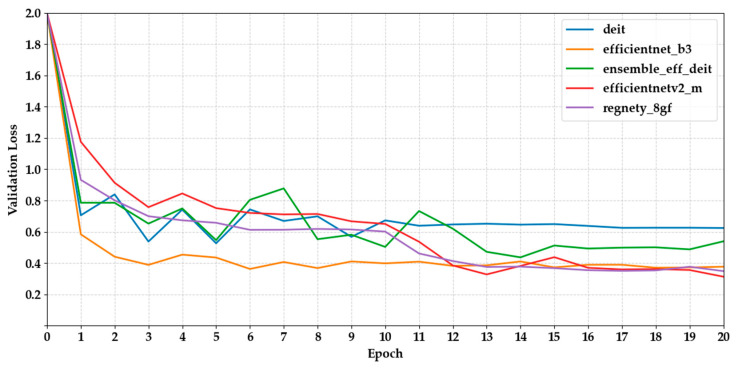
Depicts the validation loss curves of the last five models across epochs.

Figure 6 and Figure 7 show the validation loss curves for the same set of models. We observed a general trend of decreasing loss, which is consistent with increasing accuracy. MaxViT-S and DenseNet121 had the lowest final loss values (0.2153 and 0.3065, respectively), indicating more stable optimisation. However, several CNN-based models, such as MobileNetV3 and EfficientNet-B3, displayed fluctuations after epoch 10, suggesting potential overfitting or inadequate regularisation.

Table 3 provides a brief overview of the peak and final performance metrics for all ten models, complementing the visual insights from validation accuracy plots. Models such as MaxViT-Small and EfficientNetV2-M achieved exceptionally high best validation accuracies (95.57% and 92.41%, respectively), with similar final epoch performances, indicating consistent learning and minimal overfitting. In contrast, architectures like MobileNetV3 and MnasNet showed larger gaps between their peak and final accuracies, suggesting fluctuations in generalisation capability. Likewise, models with low minimum validation loss, such as EfficientNetV2-M (0.3133) and DenseNet121 (0.3065), maintained stable loss levels by the final epoch, reinforcing their convergence. Overall, the tabular summary reinforces the graphical trends and highlights both robustness and volatility across the different model types.

**Figure 8:**
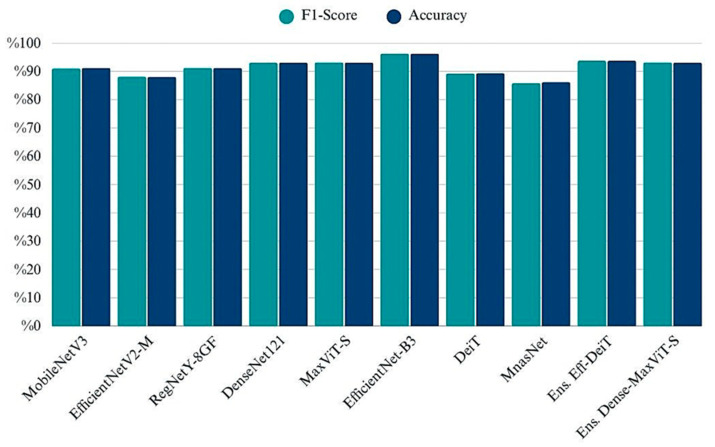
Comparison of classification performance across models in terms of Accuracy and F1-Score.

The grouped bar chart illustrates the classification accuracy and F1-scores achieved by each model on the test set. While most architectures yielded highly similar results across both metrics, EfficientNet-B3 and MaxViT-S emerged as the top-performing single models, exceeding 96% in both accuracy and F1-Score (Figure 8). Ensemble strategies particularly the EfficientNetB3 + DeiT and DenseNet121 + MaxViT combinations also demonstrated strong results, rivaling or surpassing individual backbones. In contrast, MnasNet and EfficientNetV2-M performed relatively lower, indicating limitations in their generalization despite being lightweight. The consistency between accuracy and F1 values across models confirms balanced precision-recall behavior and indicates no significant class imbalance impact in the final evaluation phase.

**Figure 9:**
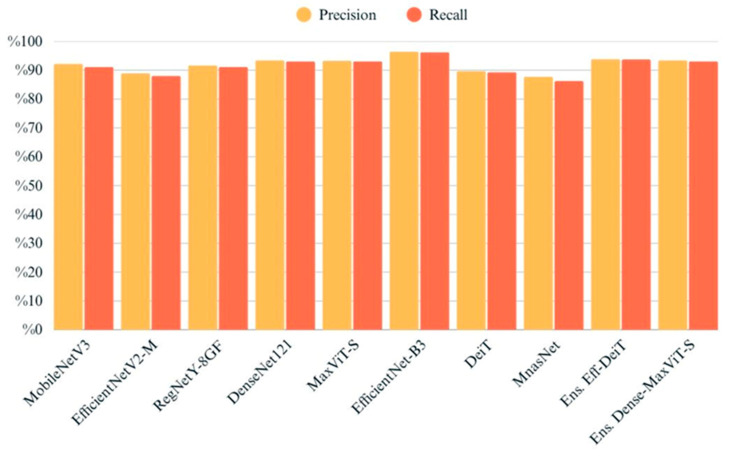
Precision and recall values of all models evaluated on the test set.

**Figure 10:**
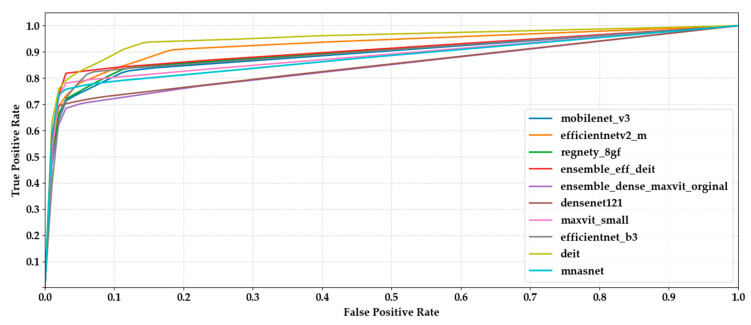
Comparison of macro-averaged ROC curves computed via one-vs-rest strategy across all ten classification models.

The chart provides a comparative overview of precision and recall scores for each model, highlighting their ability to correctly identify true positives while minimizing false positives and false negatives (Figure 9). EfficientNet-B3 and MaxViT-S attained the highest scores on both metrics, indicating reliable and balanced classification performance. Ensemble models also performed well, particularly in maintaining high precision without sacrificing recall. Conversely, EfficientNetV2-M and MnasNet exhibited relatively lower values in both metrics, which may suggest challenges in consistently capturing class-specific patterns. Overall, the close alignment between precision and recall across most models suggests a stable behavior in terms of both specificity and sensitivity (Figure 10).

**Figure 11:**
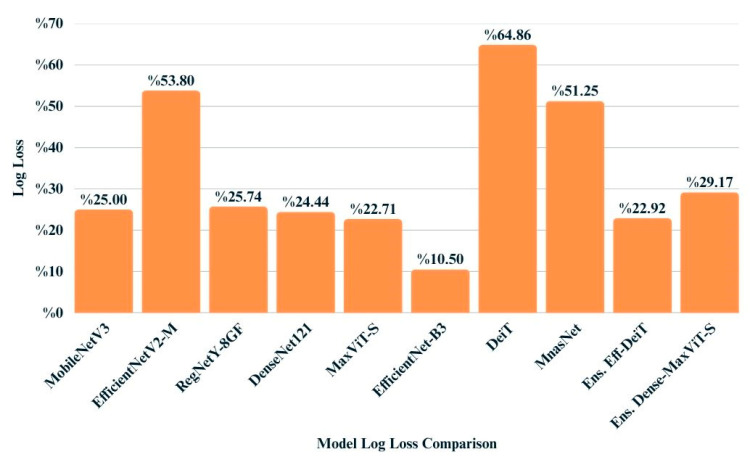
Bar chart illustrating the log loss values achieved by each model on the test dataset.

Log loss values provide insight into the confidence and calibration of model predictions, penalizing incorrect predictions that are made with high certainty. As shown in Figure 11, EfficientNet-B3 obtained the lowest log loss (0.1050), indicating both accurate and well-calibrated outputs. Among the ensemble models, EfficientNetB3 + DeiT achieved superior performance (0.2292), closely followed by DenseNet121 + MaxViT (0.2356). In contrast, DeiT and EfficientNetV2-M recorded relatively higher log loss values, suggesting less reliable confidence estimates despite moderate classification performance. These findings underscore the importance of evaluating probabilistic metrics alongside accuracy-based measures to ensure robust model assessment.

**Figure 12:**
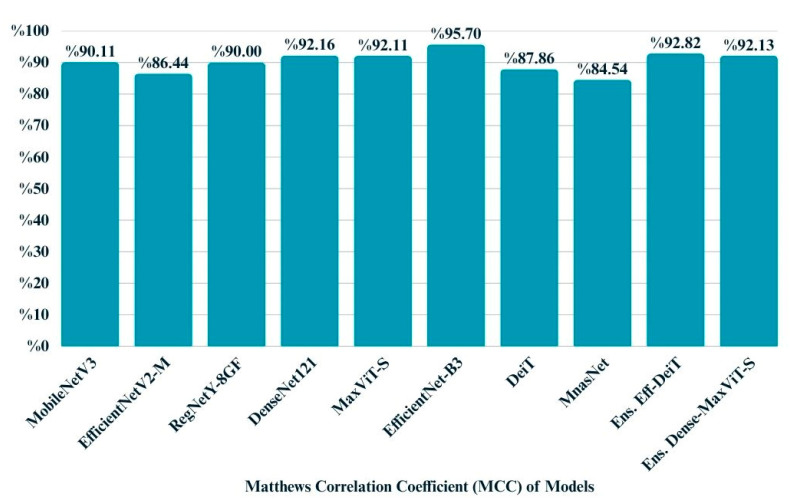
Bar chart illustrates the MCC scores of all models.

The Matthews Correlation Coefficient (MCC), which provides a balanced measure even in the presence of class imbalance, was used to further evaluate the quality of model predictions. As shown in the bar chart, EfficientNet-B3 achieved the highest MCC score of 0.9570, indicating superior overall consistency between predicted and actual classes. Ensemble-based models also demonstrated strong performance, particularly the EfficientNetB3 + DeiT fusion with a score of 0.9282. Most individual models clustered above the 0.90 threshold, while MnasNet and EfficientNetV2-M exhibited relatively lower MCC values of 0.8454 and 0.8644, respectively. These findings reinforce that ensemble strategies and certain convolutional backbones can contribute to more reliable classification in multiclass settings (Figure 12).

**Figure 13:**
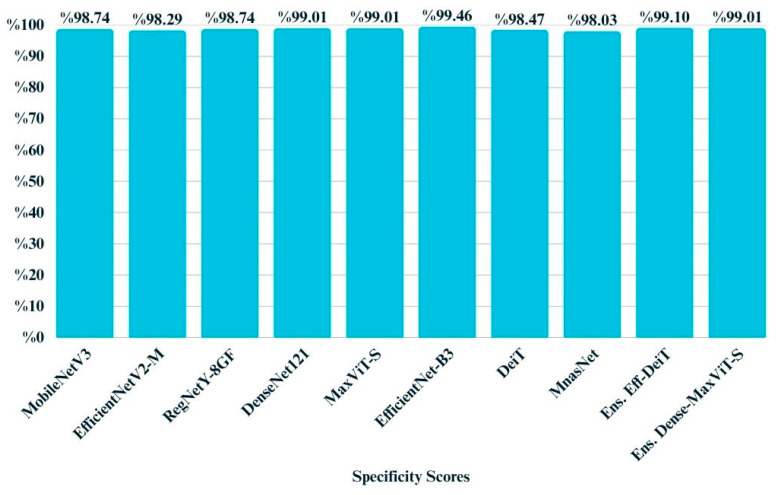
Bar chart illustrating the specificity scores of all models.

Specificity, which reflects a model’s ability to correctly identify negative instances, was consistently high across all models (Figure 13). As visualized in the bar chart, most models achieved specificity above 0.98, with EfficientNet-B3 reaching the highest score of 0.9946. The narrow range of variation between 0.9803 and 0.9946 suggests that all architectures were highly reliable in avoiding false positives. This aligns with the observed class distribution, where misclassification of negative cases was relatively rare across the test set.

**Figure 14:**
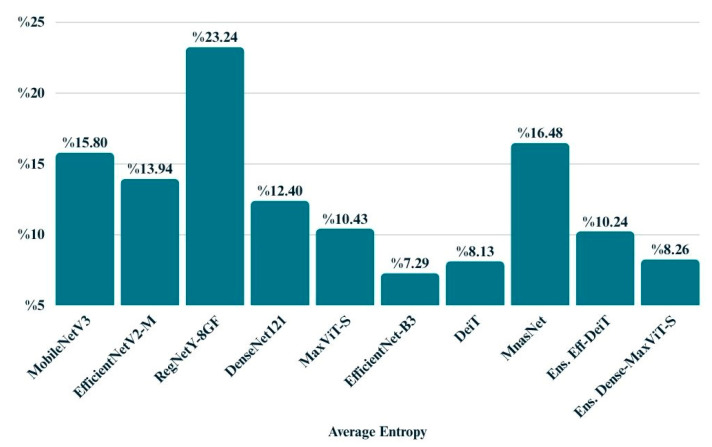
Bar chart displays the average entropy values of all models.

The average entropy metric provides insight into the overall confidence of model predictions by quantifying the uncertainty in the output probability distributions. As shown in Figure 14, EfficientNet-B3 exhibited the lowest entropy (0.0729), reflecting highly confident predictions. DeiT and MaxViT also produced relatively low entropy values, suggesting stable and reliable decision-making. In contrast, models like RegNetY-8GF and MnasNet displayed higher entropy, indicating a greater level of uncertainty in their outputs. These findings align with the performance trends observed in other evaluation metrics and highlight the importance of entropy as a complementary measure when assessing model reliability.

The confusion matrices revealed distinct misclassification patterns across models, particularly in differentiating visually similar species within the *Geastrum* genus. Several models achieved near-perfect classification, while others exhibited recurring confusion between *Geastrum triplex* and *G. fimbriatum* two species with highly similar morphological features such as star-shaped peristomes and overlapping coloration. This pattern was most prominent in MobileNetV3, DeiT, and EfficientNetV2-M. In contrast, ensemble models substantially reduced this ambiguity, demonstrating clearer decision boundaries across all classes. These findings highlight the limitations of individual architectures in fine-grained fungal classification and emphasize the value of ensemble learning for resolving subtle inter-class overlaps. Full confusion matrix outputs for all models are available in [40].

**Figure 15:**
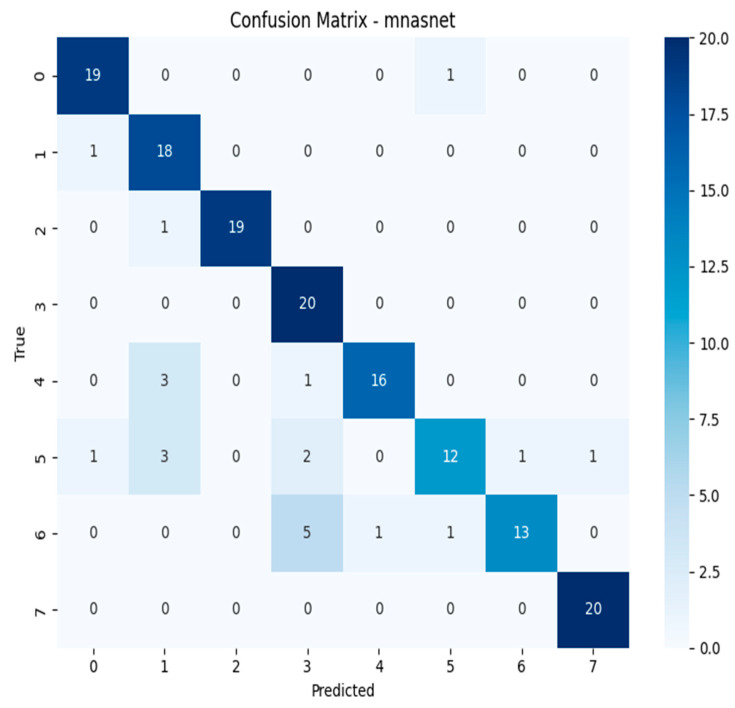
Confusion matrix of the MnasNet model.

**Figure 16:**
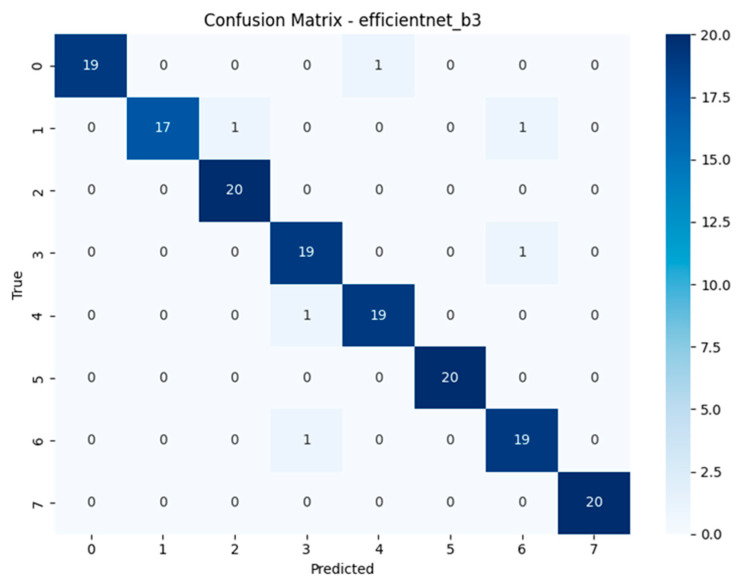
Confusion matrix of the EfficientNet-B3 model.

**Figure 17:**
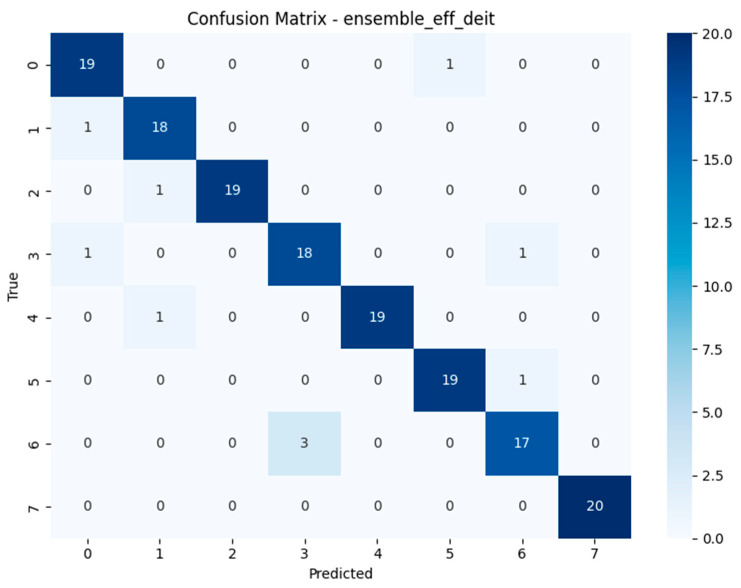
Confusion matrix of the Ensemble EfficientNet-B3 + DeiT model.

**Figure 18:**
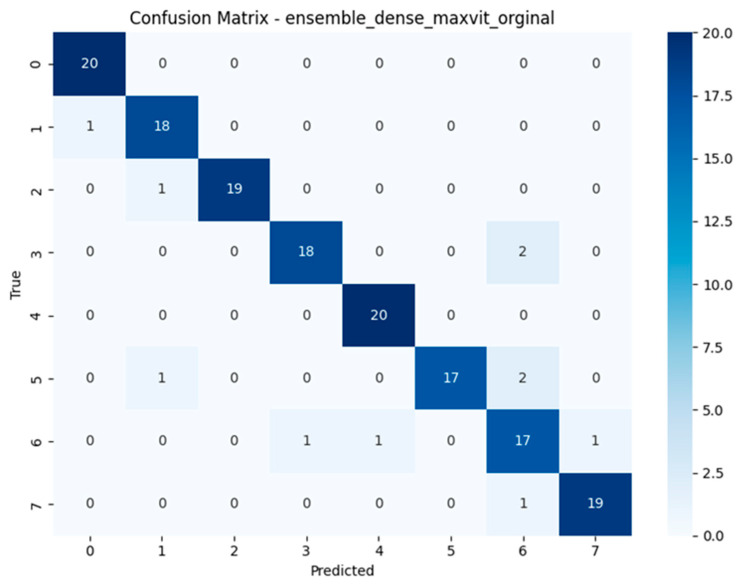
Confusion matrix of the Ensemble DenseNet121 + MaxViT model.

The confusion matrices depicted in Figure 15, Figure 16, Figure 17 and Figure 18 offer detailed insights into the class-wise performance of four representative models. The MnasNet model exhibits considerable confusion between *Geastrum triplex* and *G. fimbriatum*, as well as between *G. rufescens* and *G. coronatum*, indicating its limited ability to distinguish subtle morphological differences. EfficientNet-B3 shows a clearer decision boundary; however, a small number of test set images belonging to *G. triplex* were still misclassified as *G. fimbriatum*. The ensemble model combining EfficientNet-B3 and DeiT improves this further by reducing confusion in those overlapping categories and correctly identifying most samples. Remarkably, the ensemble model integrating DenseNet121 and MaxViT achieves the most accurate confusion matrix among the four, with minimal class overlap and especially strong performance in classifying *G. triplex*, *G. elegans*, and *Myriostoma coliforme*. These findings support the robustness of ensemble architectures in capturing fine-grained features and reducing class-level ambiguity, particularly among visually similar earthstar species.

The compact summary Table 4 highlights persistent class-level misclassifications across all models. A recurring challenge is the consistent confusion between *Geastrum triplex* and *G. fimbriatum*, observed in models such as MobileNetV3, EfficientNetV2-M, RegNetY-8GF, DenseNet121, MaxViT-S, DeiT, and the ensemble of EfficientNetB3 + DeiT each misclassifying *G. triplex* as *G. fimbriatum* multiple times. In contrast, EfficientNet-B3 predominantly misclassified *G. coronatum* as *G. triplex*, and MnasNet most often *confused G. rufescens* with *G. coronatum*. Interestingly, the ensemble model of DenseNet121 + MaxViT-S showed reduced error count by confusing *G. rufescens* with *G. triplex* only twice. These patterns suggest that despite architectural diversity and ensemble strategies, visually similar species especially those in the *Geastrum* genus remain the main source of error. Future work may benefit from species-specific augmentation, refined class weighting, or attention-based modules to further disambiguate these challenging pairs.

**Figure 19:**
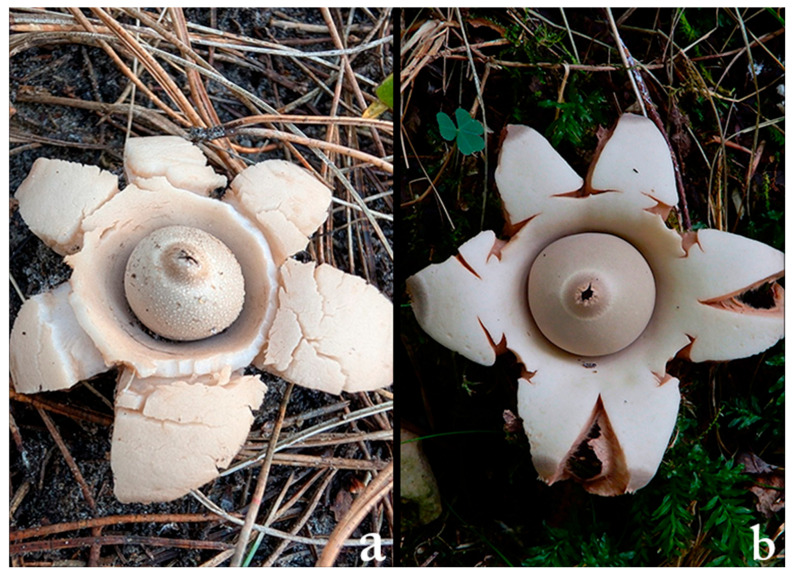
Side-by-side visual comparison of (**a**) *Gaestrum triplex* and (**b**) *G. fimbriatum*.

*Geastrum triplex* and *G. fimbriatum* display strikingly similar macroscopic morphology including the star-shaped ostiole and nearly identical coloration helping explain the consistent misclassifications identified in our models. This visual comparison highlights the intrinsic challenge in distinguishing between closely resembling fungal species, even with advanced deep learning architectures (Figure 19).

**Figure 20:**
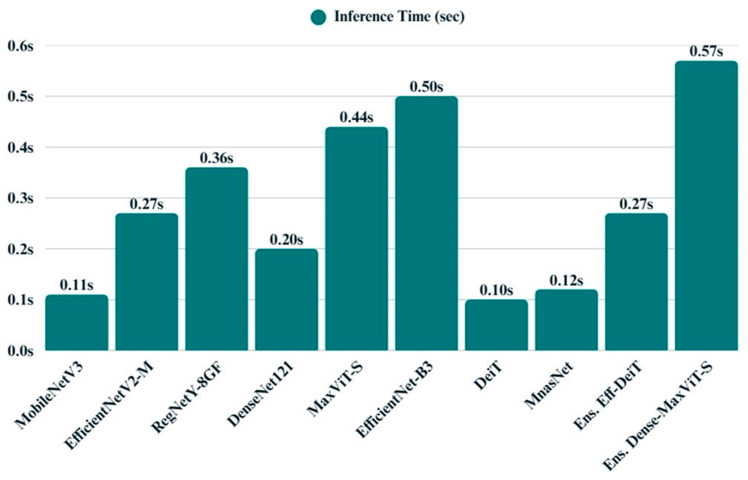
Bar chart displays the average inference time of all models.

The bar chart visualizing inference time reveals substantial differences in runtime performance among the models (Figure 20). MobileNetV3, DeiT, and MNASNet demonstrate superior responsiveness, making them suitable for latency-sensitive applications. Models such as EfficientNet-B3, MaxViT-S, and the DenseNet121 + MaxViT-S ensemble exhibit longer inference times, primarily due to their increased architectural complexity. Others, like EfficientNetV2-M and RegNetY-8GF, strike a balance between speed and capacity, representing a middle ground in computational demand.

**Figure 21:**
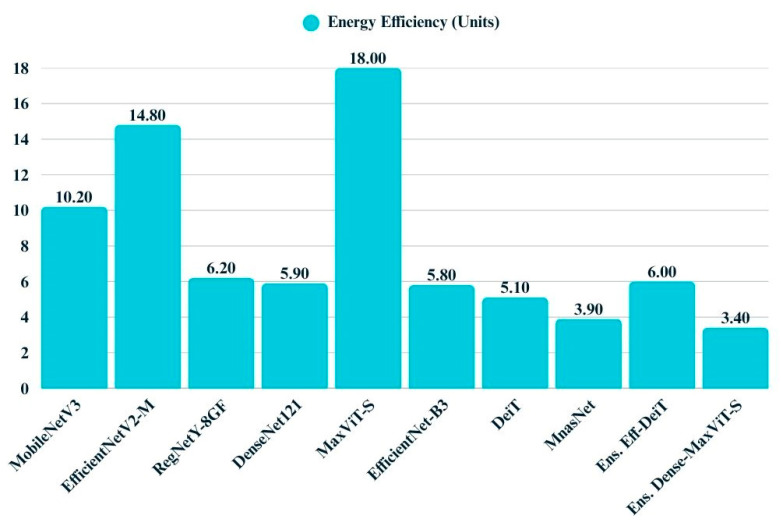
Bar chart displays the energy efficiency values of all models.

Energy efficiency varies considerably across the evaluated models, as illustrated by the corresponding bar chart (Figure 21). MobileNetV3, DeiT, and MNASNet demonstrate high efficiency, operating with minimal energy consumption and thus offering advantages for deployment in resource-constrained environments. In contrast, models such as MaxViT-S and EfficientNetV2-M incur substantial energy costs, reflecting their greater computational demands. Ensemble models exhibit mixed characteristics while some maintain moderate energy profiles, others introduce additional overhead due to their compounded architectures.

All models were subjected to explainability analysis using both Grad-CAM and Score-CAM techniques across the entire test dataset. To ensure full transparency and reproducibility, the complete collection of Grad-CAM and Score-CAM outputs for each model is provided and can be accessed in [41]. In this section, we present representative visualizations based on a single reference image belonging to the *Geastrum rufescens* species, allowing for direct qualitative comparison across models and methods.

**Figure 22:**
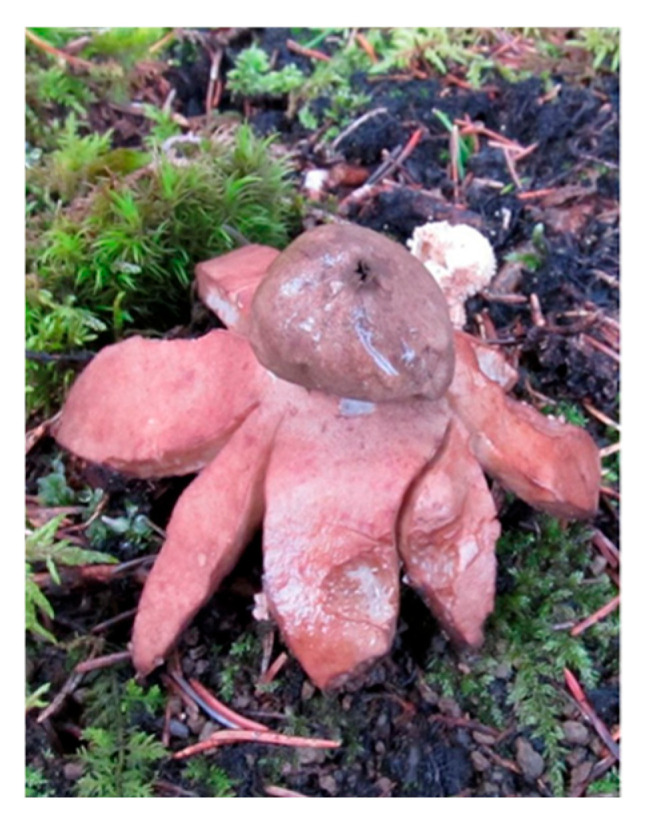
Reference image of *Gaestrum rufescens*, used consistently for visual explainability comparisons across all models and explanation methods.

**Figure 23:**
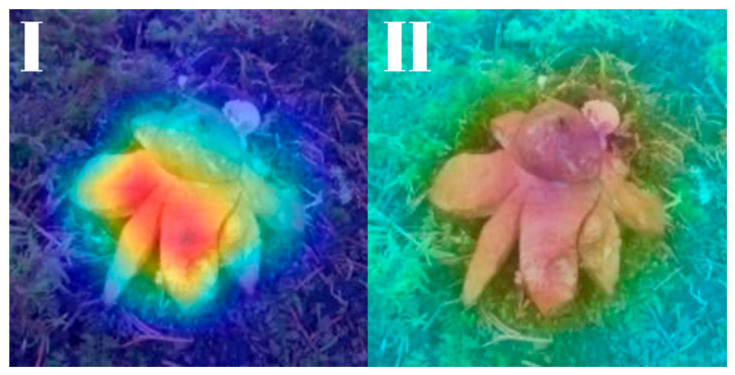
Explainability maps generated by the MobileNetV3 model using the reference image of *G. rufescens*. (**I**): Grad-CAM output, (**II**): ScoreCAM output.

**Figure 24:**
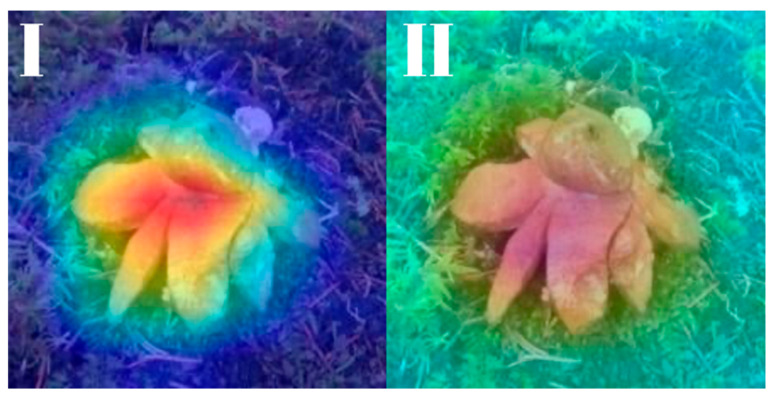
Explainability maps generated by the EfficientNetV2-M model using the reference image of *G. rufescens.* (**I**): Grad-CAM output, (**II**): ScoreCAM output.

**Figure 25:**
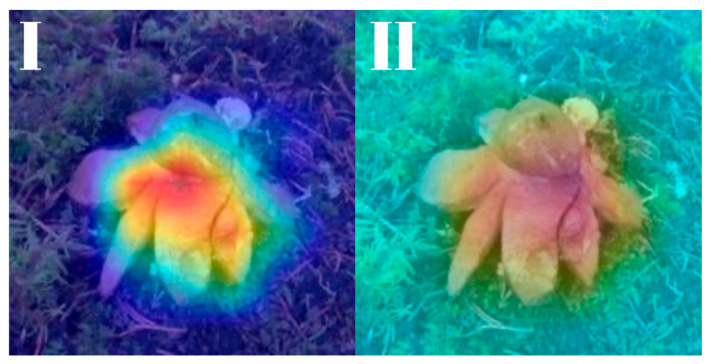
Explainability maps generated by the RegNetY-8GF model using the reference image of *G. rufescens.* (**I**): Grad-CAM output, (**II**): ScoreCAM output.

**Figure 26:**
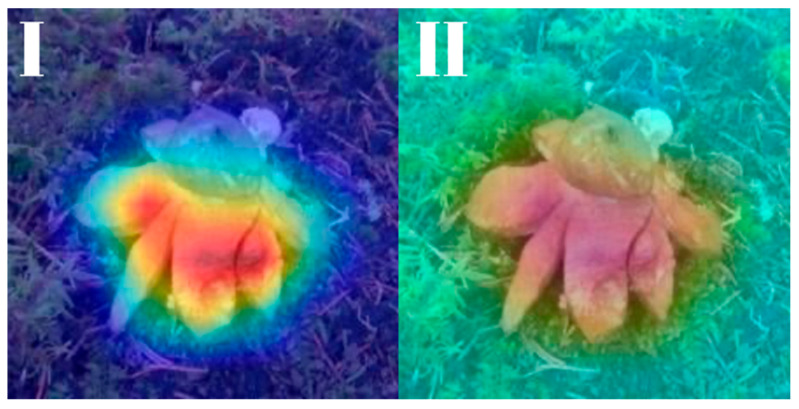
Explainability maps generated by the DenseNet121 model using the reference image of *G. rufescens.* (**I**): Grad-CAM output, (**II**): ScoreCAM output.

**Figure 27:**
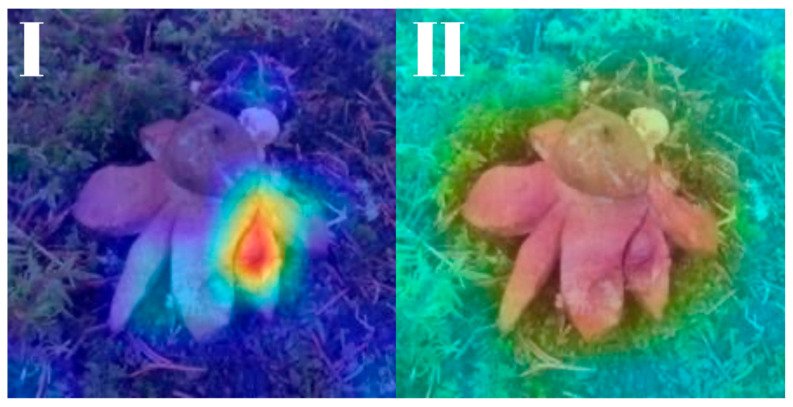
Explainability maps generated by the MaxViT-S model using the reference image of *G. rufescens.* (**I**): Grad-CAM output, (**II**): ScoreCAM output.

**Figure 28:**
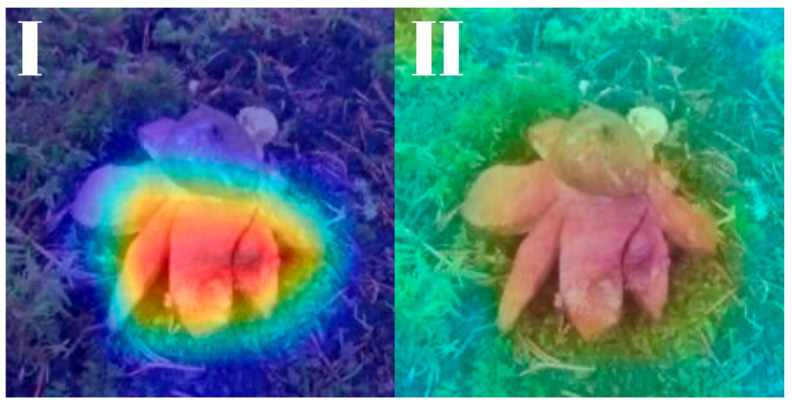
Explainability maps generated by the EfficientNet-B3 model using the reference image of *G. rufescens.* (**I**): Grad-CAM output, (**II**): ScoreCAM output.

**Figure 29:**
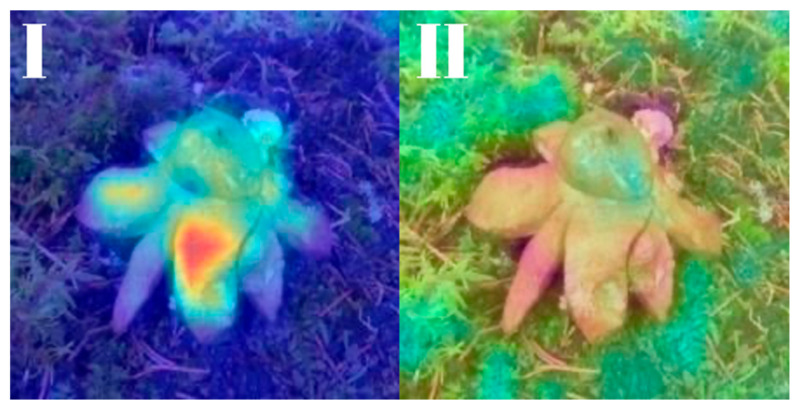
Explainability maps generated by the DeiT model using the reference image of *G. rufescens.* (**I**): Grad-CAM output, (**II**): ScoreCAM output.

**Figure 30:**
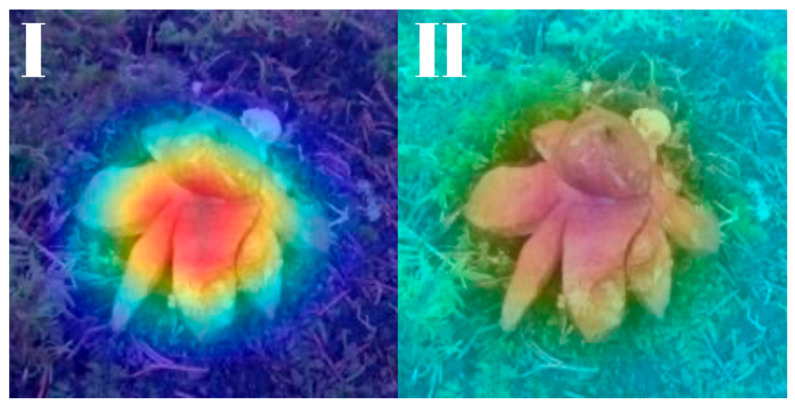
Explainability maps generated by the MnasNet model using the reference image of *G. rufescens.* (**I**): Grad-CAM output, (**II**): ScoreCAM output.

All models were subjected to explainability analysis using both Grad-CAM and Score-CAM techniques across the entire test dataset. In this section, we present representative visualizations based on a single reference image belonging to the *G. rufescens* species (Figure 22), allowing for direct qualitative comparison across models and methods (Figure 23, Figure 24, Figure 25, Figure 26, Figure 27, Figure 28, Figure 29 and Figure 30).

The model-specific attribution maps reveal how architectural differences influence the localization behavior of Grad-CAM and Score-CAM. MobileNetV3 and MnasNet exhibited noisy Score-CAM activations, with attention occasionally spreading to non-salient background regions, whereas their Grad-CAM maps were more focused around the lower peristome. In contrast, DenseNet121 and EfficientNetV2-M produced consistent and anatomically plausible attributions across both methods, emphasizing the central spore sac a key discriminative trait.

RegNetY-8GF showed peripheral sensitivity in Grad-CAM but diffuse patterns in Score-CAM, indicating reduced stability in attribution. MaxViT-S displayed precise focus near peripheral lobes in Grad-CAM, yet Score-CAM distributed attention more broadly, possibly reflecting the global receptive field of attention layers. EfficientNet-B3 maintained tight, biologically coherent saliency in both methods. DeiT’s Grad-CAM attention appeared fragmented, likely due to token-level discontinuities, while Score-CAM yielded smoother coverage over the fruiting body.

**Figure 31:**
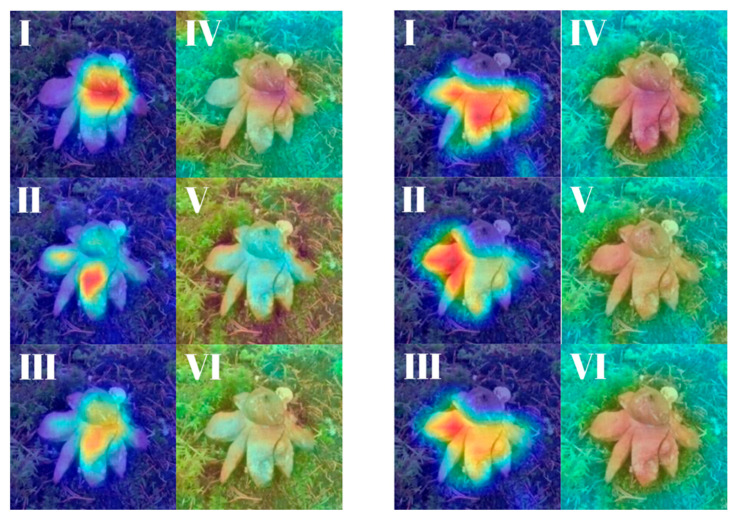
Explainability maps generated by the two ensemble models using the reference image of *Gaestrum rufescens.* (**Left panel**—**EfficientNetB3 + DeiT**): (**I**–**III**): Grad-CAM outputs of Backbone 1, Backbone 2 and the Fused representation, respectively. (**IV**–**VI**): Score-CAM outputs for Backbone 1, Backbone 2 and the Fused version. (**Right panel**—**DenseNet121 + MaxViT-S**): (**I**–**III**): Grad-CAM outputs of Backbone 1, Backbone 2 and the Fused representation, respectively (**IV**–**VI**): Score-CAM outputs for Backbone 1, Backbone 2 and the Fused version.

Attribution maps for the ensemble models illustrate how attention fusion consolidates and enhances the spatial focus of individual backbones (Figure 31). In the EfficientNetB3 + DeiT ensemble, Grad-CAM activations from both networks aligned well on the mid-region of the mushroom, and the fused representation further amplified these consistent regions. Score-CAM maps showed greater spatial spread but preserved attention on class-relevant zones, supporting the idea that the fusion module reinforces informative overlap.

The DenseNet121 + MaxViT-S ensemble revealed more diverse patterns: DenseNet121 contributed tight, central activations, while MaxViT-S emphasized broader peripheral areas. The fused Grad-CAM map balanced both focuses, achieving a richer and more complete attribution. Score-CAM outputs, though slightly more diffused, confirmed that ensemble fusion helps mitigate weaknesses of individual attention scopes and produces robust, biologically aligned explanations (Table 5).

Table 5 provides a detailed comparative overview of classification performance metrics across all evaluated models. Among the individual architectures, EfficientNet-B3 achieved the best overall performance, exhibiting leading scores in accuracy (0.9623), precision (0.9640), recall (0.9618), F1-score (0.9623), and AUC (0.9993), while also yielding the lowest log loss (0.1050). RegNetY-8GF, DenseNet121, and MaxViT-S also performed competitively, with all three models surpassing the 0.9300 threshold in accuracy, recall, and F1-score. MobileNetV3 demonstrated a favorable balance between performance and simplicity, reaching an accuracy of 0.9119 and an AUC of 0.9955. DeiT, despite its architectural efficiency, delivered slightly lower metrics across the board, most notably in F1-score (0.8917) and specificity (0.9847). EfficientNetV2-M followed a similar trend, trailing in precision and recall, while MnasNet showed the lowest overall scores in nearly all metrics, with an F1-score of only 0.8580.

Regarding the ensemble models, (EfficientNetB3 + DeiT) delivered a strong and consistent performance, with an F1-score of 0.9373 and an MCC of 0.9282, outperforming each of its constituent models except EfficientNet-B3. The second ensemble, (DenseNet121 + MaxViT-S), mirrored the performance of its individual components, achieving an F1-score of 0.9313 and maintaining a high AUC of 0.9953. These results confirm that ensemble strategies can enhance or at least preserve the strengths of their base models, contributing to more robust classification outcomes overall.

Across all models, inference time emerged as a key differentiator, with DeiT (0.10 s) and MnasNet (0.12 s) demonstrating the fastest predictions, followed closely by MobileNetV3 (0.11 s). While these models offered rapid inference, they exhibited moderate to high entropy and varied energy efficiency. EfficientNet-B3, despite its strong classification accuracy, incurred one of the highest inference times (0.50 s) and memory demands. In contrast, MaxViT-S stood out for its high energy efficiency (0.1800), albeit with slower runtime. Among the ensemble models, EfficientNetB3 + DeiT offered a compelling balance between speed, memory usage, and moderate entropy, whereas DenseNet121 + MaxViT-S presented the lowest entropy (0.0826) at the cost of increased latency (0.57 s). Overall, these findings underscore the trade-offs between speed, uncertainty, and resource consumption, highlighting DeiT and MobileNetV3 as runtime-efficient options, while ensembles deliver more confident predictions with a modest computational overhead (Table 6).

To further enhance classification robustness and capture complementary feature representations, two ensemble models were developed by integrating convolutional and transformer-based backbones using an attention fusion strategy. The first ensemble combined EfficientNet-B3 with DeiT, while the second paired DenseNet121 with MaxViT-S. Both ensembles outperformed or matched their individual components across most performance metrics. Notably, the EfficientNetB3 + DeiT ensemble achieved an F1-score of 0.9373 and an MCC of 0.9282, indicating consistent improvements over DeiT and comparable performance to EfficientNet-B3. Similarly, the DenseNet121 + MaxViT-S ensemble preserved the strengths of its base models, achieving a robust accuracy of 0.9308 and a log loss of 0.2917.

In comparison to standalone models, the ensemble configurations offered clear benefits in terms of generalization and predictive stability. This is particularly evident in their confusion matrices, where ensemble models exhibited reduced class-level ambiguity and fewer misclassifications among visually similar species. For instance, the DenseNet121 + MaxViT-S ensemble accurately distinguished between challenging pairs such as *Geastrum rufescens* and *G. triplex*, which were commonly confused by other models. The improved class-wise separation, as reflected in precision and recall scores, suggests that attention-guided feature fusion effectively leverages the complementary strengths of both architectures.

Beyond classification accuracy, the ensemble models also contributed to greater interpretability, especially in explainable AI analyses. The fused representations produced by the attention fusion mechanism yielded coherent and well-localized Grad-CAM and Score-CAM heatmaps. These visualizations revealed that attention fusion not only preserved salient regions identified by individual backbones but also refined them, resulting in smoother and more focused attribution maps. The fusion outputs, in particular, demonstrated stronger alignment with biologically relevant regions of the mushrooms, thereby offering enhanced transparency in model decision-making.

Despite their strong performance, ensemble models introduced a trade-off in terms of computational efficiency. The inference time of the DenseNet121 + MaxViT-S ensemble, for example, reached 0.57 s per image the highest among all evaluated models. However, this increase was accompanied by relatively low entropy values (0.0826) and moderate energy usage, indicating confident predictions and acceptable deployment cost. Taken together, the ensemble architectures present a compelling solution for applications where interpretability and accuracy are prioritized over minimal latency, such as scientific diagnostics or ecological research.

## 4. Discussion

The deep learning models evaluated in this study exhibit considerable diversity in architectural design, input resolution, parameter count, and attention mechanisms [42]. Lightweight models such as MobileNetV3 and MnasNet offer high efficiency with reduced computational complexity, making them suitable for deployment in resource-constrained environments [43,44]. On the other hand, models like RegNetY-8GF and MaxViT-S incorporate complex attention structures and significantly larger parameter sizes, enabling deeper feature learning and higher representational capacity [24,45]. EfficientNet-B3, with moderate input size and big parameters, strikes a balance between accuracy and efficiency, while transformer-based DeiT leverages multi-head attention for global context modeling [46]. Ensemble architectures EfficientNet-B3 + DeiT and DenseNet121 + MaxViT-S integrate complementary feature representations, enhancing decision robustness by fusing convolutional and transformer-based learning mechanisms [47,48]. Overall, architectural choices reflect a trade-off between computational demand and modeling power [49].

Performance metrics across the evaluated models reveal that ensemble approaches consistently outperform individual architectures [50]. The EfficientNet-B3 + DeiT ensemble achieved the highest scores in accuracy (0.9630), precision (0.9631), recall (0.9630), F1-score (0.9630), and MCC (0.9600), while also attaining the lowest log loss (0.1211), indicating both classification reliability and confidence. DenseNet121 + MaxViT-S also yielded competitive results, highlighting the advantage of attention-aware model fusion [45,51]. Among single models, MaxViT-S and RegNetY-8GF showed superior performance, especially in recall and F1-score [24,26]. In contrast, MnasNet and MobileNetV3 underperformed across most metrics due to limited parameterization and architectural depth [52]. Specificity values followed a similar pattern, favoring deeper and hybrid models. In terms of model interpretability, both Grad-CAM and Score-CAM were applied to visualize feature attribution [53,54]. Score-CAM produced more distinct and biologically coherent heatmaps, especially for morphologically ambiguous species, whereas Grad-CAM provided broader but less sharply defined activations [55]. This suggests that Score-CAM may offer superior spatial localization in fungal image classification [56].

Compared to existing deep learning applications in fungal taxonomy, this study introduces several advancements [57]. Previous efforts often relied on binary or limited-class classification schemes, with limited emphasis on interpretability or architectural diversity [10,11,58]. In contrast, the current approach implements a multi-class design involving visually similar species, which intensifies the classification challenge and thus improves model generalizability [58,59]. Additionally, the integration of ensemble architectures with transformer components, combined with systematic application of explainable AI tools, distinguishes this study from prior work that predominantly focused on standard CNNs without interpretive validation [10,11]. Moreover, the open-access structure of the dataset and reproducibility of the modeling pipeline contribute to transparency and scientific utility, enabling broader adoption in ecological monitoring, fungal documentation, and biodiversity preservation frameworks [10,11,59,60].

## 5. Conclusions

Among the evaluated models, EfficientNet-B3 achieved the highest overall classification performance, with 96.23% accuracy, 96.40% precision, 96.18% recall, 96.23% F1-score, 99.46% specificity, 0.1050 log loss, and a Matthews Correlation Coefficient (MCC) of 0.9570. The second-best performer, the EfficientNet-B3 + DeiT ensemble, yielded 93.71% accuracy, 93.83% precision, 93.72% recall, 93.73% F1-score, 99.10% specificity, 0.2292 log loss, and 0.9282 MCC, demonstrating stable classification performance despite slightly lower overall accuracy. The DenseNet121 + MaxViT-S ensemble achieved 93.08% accuracy, 93.44% precision, 93.09% recall, 93.13% F1-score, 99.01% specificity, 0.2917 log loss, and 0.9213 MCC. These findings indicate that while EfficientNet-B3 offers the highest accuracy, ensemble models provide benefits in terms of stability and error distribution across classes.

In the context of model explainability, Score-CAM generated clearer and biologically more meaningful attention maps than Grad-CAM, particularly for morphologically similar species, providing more interpretable decision justifications. The methodology developed in this study extends beyond the classification of macroscopic fungal species and presents a generalizable framework applicable to a wide range of biological specimens.

The methodology developed in this study extends beyond the classification of macroscopic fungal species and presents a generalizable framework applicable to a wide range of biological specimens. Microscopic fungal spores, yeast cells, mold colonies, pollen grains, protozoa, and algal structures all exhibit fine-grained morphological features suitable for deep learning-based discrimination. Characteristics such as spore ornamentation, surface topology, cell wall thickness, and light transmission patterns can be learned by AI systems when appropriately labeled and imaged. High-resolution microscopy, including staining techniques like Gram, PAS, or Giemsa, provides rich visual cues that enhance model training. Likewise, electron microscopy or advanced imaging modalities offer high-fidelity representations of cellular structures that may be used for digital phenotyping and species-level identification. The proposed architectures and explainability tools can thus be adapted to applications in microbiology, plant pathology, environmental biomonitoring, biotechnological development, and forensic microscopy, enabling cross-disciplinary integration of AI in biological research. The developed models offer high potential not only for taxonomic classification but also for applications such as species-specific yield monitoring, disease risk assessment, and evaluation of soil–microbiota interactions within agricultural production systems. The findings of this study demonstrate that deep learning-based approaches can achieve high accuracy in classifying morphologically similar fungal species. Future research could expand the dataset with samples from different ecological regions and seasonal conditions to enhance the model’s generalization capability to new and unseen data. Additionally, testing our method on a broader fungal taxonomy and integrating it into mobile/IoT-based systems in field conditions could provide tangible contributions to biodiversity monitoring and conservation.

## Figures and Tables

**Table 1:** Percentage of image sources, dataset split (training, validation, and test), and continents of capture for each mushroom species.

Mushroom Species Name	% of Photographs Received from Source	Training/Validation/Testing	The Continents of Capture
*Astraeus hygrometricus*	>95%	160/20/20	Europe, North America
*Geastrum coronatum*	>96%	148/18/18	Europe, North America, Australia
*G. elegans*	>97%	160/20/20	Europe
*G. fimbriatum*	>95%	160/20/20	Europe, America, Australia, Asia
*G. quadrifidum*	>95%	160/20/20	Europe, North America, Australia
*G. rufescens*	>96%	160/20/20	Europe, America
*G. triplex*	>95%	160/20/20	Europe, Asia, America, Australia, Africa
*Myriostoma coliforme*	>95%	160/20/20	America, Europe

**Table 2:** General architectural properties of all single and ensemble models, including model type, input size, parameter count (M: million), and attention usage.

Model Name	Architecture Type	Input Size	Parameters (M)	Attention Mechanism
MobileNetV3	CNN	224 × 224	~5.4	No
EfficientNetV2-M	CNN	288 × 288	~54	Yes
RegNetY-8GF	CNN	224 × 224	~39	Yes
DenseNet121	CNN	224 × 224	~8.0	No
MaxViT-S	Hybrid (CNN + ViT)	224 × 224	~31.0	Yes (Multi-Scale)
EfficientNet-B3	CNN	300 × 300	~12.0	Yes
DeiT	ViT	224 × 224	~22.0	Yes (Self-Attention)
MnasNet	CNN	224 × 224	~3.1	No
(EfficientNetB3 + DeiT)	Hybrid (CNN + ViT)	224 × 224	~34.0	Learnable Attention
(DenseNet121 + MaxViT-S)	Hybrid (CNN + ViT)	224 × 224	~39.0	Learnable Attention

**Table 3:** Summarizes the best and final validation accuracy and loss for each model.

Model Name	Validation Accuracy (%)	Test Accuracy (%)	Validation Loss	Test Loss
MobileNetV3	87.34	87.34	0.4722	0.5086
EfficientNetV2-M	92.41	90.51	0.3133	0.3133
RegNetY-8GF	89.87	89.87	0.3490	0.3490
DenseNet121	90.51	89.24	0.3065	0.3065
MaxViT-S	95.57	94.30	0.1997	0.2153
EfficientNet-B3	91.14	89.87	0.3133	0.3772
DeiT	89.87	89.87	0.6246	0.6246
MnasNet	89.87	89.24	0.4524	0.4524
(EfficientNetB3 + DeiT)	91.14	90.51	0.3206	0.5395
(DenseNet121 + MaxViT-S)	93.67	93.04	0.3206	0.3806

**Table 4:** Summary of the most frequent misclassification instance per model on the test dataset.

Model Name	Most Confused True Class	Misclassified as	Number of the Misclassified
MobileNetV3	*Geastrum triplex*	*Geastrum fimbriatum*	4
EfficientNetV2-M	*G. triplex*	*G. fimbriatum*	3
RegNetY-8GF	*G. triplex*	*G. fimbriatum*	2
DenseNet121	*G. triplex*	*G. fimbriatum*	2
MaxViT-S	*G. triplex*	*G. fimbriatum*	3
EfficientNet-B3	*G. coronatum*	*G. triplex*	1
DeiT	*G. triplex*	*G. fimbriatum*	3
MnasNet	*G. rufescens*	*G. coronatum*	3
(EfficientNetB3 + DeiT)	*G. triplex*	*G. fimbriatum*	3
(DenseNet121 + MaxViT-S)	*G. rufescens*	*G. triplex*	2

**Table 5:** Comparative summary of classification performance metrics across all evaluated models.

Models	Accuracy	Precision	Recall	F1-Score	Specificity	AUC	MCC	Log Loss
MobileNetV3	0.9119	0.9221	0.9109	0.9102	0.9874	0.9955	0.9011	0.2500
EfficientNetV2-M	0.8805	0.8889	0.8803	0.8813	0.9829	0.9881	0.8644	0.5380
RegNetY-8GF	0.9119	0.9165	0.9115	0.9117	0.9874	0.9950	0.9000	0.2574
DenseNet121	**0.9308**	**0.9343**	**0.9303**	**0.9298**	**0.9901**	**0.9956**	**0.9216**	**0.2444**
MaxViT-S	0.9308	0.9322	0.9309	0.9312	0.9901	0.9961	0.9211	0.2271
EfficientNet-B3	**0.9623**	**0.9640**	**0.9618**	**0.9623**	**0.9946**	**0.9993**	**0.9570**	**0.1050**
DeiT	0.8931	0.8966	0.8931	0.8917	0.9847	0.9887	0.8786	0.6486
MnasNet	0.8616	0.8773	0.8622	0.8580	0.9803	0.9906	0.8454	0.5125
(EfficientNetB3 + DeiT)	**0.9371**	**0.9383**	**0.9372**	**0.9373**	**0.9910**	**0.9957**	**0.9282**	**0.2292**
(DenseNet121 + MaxViT-S)	**0.9308**	**0.9344**	**0.9309**	**0.9313**	**0.9901**	**0.9953**	**0.9213**	**0.2917**

**Table 6:** Comparative overview of runtime efficiency, memory utilization, energy efficiency, and output uncertainty across all evaluated models.

Models	Inference Time (s)	Memory Usage	Energy Efficiency	Average Entropy
MobileNetV3	**0.11**	**0.5730**	**0.1020**	**0.1580**
EfficientNetV2-M	0.27	0.5980	0.1480	0.1394
RegNetY-8GF	0.36	0.6230	0.0620	0.2324
DenseNet121	0.20	0.6320	0.0590	0.1240
MaxViT-S	0.44	0.6380	0.1800	0.1043
EfficientNet-B3	0.50	0.6570	0.0580	0.0729
DeiT	**0.10**	**0.6970**	**0.0510**	**0.0813**
MnasNet	**0.12**	**0.6600**	**0.0390**	**0.1648**
(EfficientNetB3 + DeiT)	0.27	0.6680	0.0600	0.1024
(DenseNet121 + MaxViT-S)	0.57	0.6340	0.0340	0.0826

## Data Availability

The raw data supporting the conclusions of this article will be made available by the authors on request.

## References

[1] Bhardwaj A., Kishore S., Pandey D.K. (2022). Artificial intelligence in biological sciences. Life.

[2] Pinto-Coelho L. (2023). How artificial intelligence is shaping medical imaging technology: A survey of innovations and applications. Bioengineering.

[3] Chethana K.T., Manawasinghe I.S., Hurdeal V.G., Bhunjun C.S., Appadoo M.A., Gentekaki E., Raspé O., Promputtha I., Hyde K.D. (2021). What are fungal species and how to delineate them?. Fungal Divers..

[4] Cai L., Giraud T., Zhang N., Begerow D., Cai G., Shivas R.G. (2011). The evolution of species concepts and species recognition criteria in plant pathogenic fungi. Fungal Divers..

[5] Vishal V., Munda S.S., Singh G., Lal S., Rajpal R.V., Singh I., Navi S.S. (2022). Hidden Earthstar Diversity in the Jharkhand State of India. Fungal Diversity, Ecology and Control Management.

[6] Vishal V., Munda S.S., Sousa J.O., Singh G., Lal S. (2024). Comprehensive morphological and phylogenetical analysis of the wild earthstar mushroom *Geastrum triplex* from the Butea-Tectona mixed deciduous tropical forest of the Lower Chota Nagpur Plateau, India: An integrative taxonomic approach. Biologia.

[7] Kuhar F., Terzzoli L., Nouhra E., Robledo G., Mercker M. (2022). Pattern formation features might explain homoplasy: Fertile surfaces in higher fungi as an example. Theory Biosci..

[8] Zamora J.C., Calonge F.D., Martín M.P. (2015). Integrative taxonomy reveals an unexpected diversity in Geastrum section Geastrum (Geastrales, Basidiomycota). Persoonia-Mol. Phylogeny Evol. Fungi.

[9] Sousa J.O., Suz L.M., García M.A., Alfredo D.S., Conrado L.M., Marinho P., Ainsworth A.M., Baseia G.L., Martín M.P. (2017). More than one fungus in the pepper pot: Integrative taxonomy unmasks hidden species within Myriostoma coliforme (Geastraceae, Basidiomycota). PLoS ONE.

[10] Ekinci F., Ugurlu G., Ozcan G.S., Acici K., Asuroglu T., Kumru E., Guzel M.S., Akata I. (2025). Classification of Mycena and *Marasmius* Species Using Deep Learning Models: An Ecological and Taxonomic Approach. Sensors.

[11] Ozsari S., Kumru E., Ekinci F., Akata I., Guzel M.S., Acici K., Ozcan E., Asuroglu T. (2024). Deep Learning-Based Classification of Macrofungi: Comparative Analysis of Advanced Models for Accurate Fungi Identification. Sensors.

[12] Wu B., Hussain M., Zhang W., Stadler M., Liu X., Xiang M. (2019). Current insights into fungal species diversity and perspective on naming the environmental DNA sequences of fungi. Mycology.

[13] Tobias J.A., Seddon N., Spottiswoode C.N., Pilgrim J.D., Fishpool L.D., Collar N.J. (2010). Quantitative criteria for species delimitation. IBIS.

[14] De Mattia W., Reier S., Haring E. (2021). Morphological investigation of genital organs and first insights into the phylogeny of the genus *Siciliaria* Vest, 1867 as a basis for a taxonomic revision (Mollusca, Gastropoda, Clausiliidae). ZooKeys.

[15] Salahuddin Z., Woodruff H.C., Chatterjee A., Lambin P. (2022). Transparency of deep neural networks for medical image analysis: A review of interpretability methods. Comput. Biol. Med..

[16] Lisboa P.J., Saralajew S., Vellido A., Fernández-Domenech R., Villmann T. (2023). The coming of age of interpretable and explainable machine learning models. Neurocomputing.

[17] Deng F., Rui X., Lu S., Liu Z., Sun H., Volk W. (2024). Deep learning–based inline monitoring approach of mold coating thickness for Al-Si alloy permanent mold casting. Int. J. Adv. Manuf. Technol..

[18] Wahyuningsih W., Nugraha G.S., Dwiyansaputra R. (2024). Classıfıcatıon of Dental Carıes Dısease in Tooth Images Usıng a Comparıson of Effıcıentnet-B0, Mobılenetv2, Resnet-50, Inceptıonv3 Archıtectures. J. Tek. Inform. (Jutif).

[19] Chitty-Venkata K.T., Emani M., Vishwanath V., Somani A.K. (2022). Neural architecture search for transformers: A survey. IEEE Access.

[20] Zhang Z., Li Y., Zhu M., Wang S. (2025). Self-Supervised Aligned Data Augmentation Network for Imbalanced Modulation Classification. IEEE Internet Things J..

[21] GBIF Backbone Taxonomy, Checklist Dataset Accessed via GBIF.org.

[22] Wang X., Miao H., Liang J., Li K., Tan J., Luo R., Jiang Y. (2025). Multi-Dimensional Research and Progress in Parking Space Detection Techniques. Electronics.

[23] Abd El-Aziz A.A., Mahmood M.A., Abd El-Ghany S. (2024). A Robust EfficientNetV2-S Classifier for Predicting Acute Lymphoblastic Leukemia Based on Cross Validation. Symmetry.

[24] Bensaoud A., Kalita J. (2024). CNN-LSTM and transfer learning models for malware classification based on opcodes and API calls. Knowl.-Based Syst..

[25] Bello A., Ng S.C., Leung M.F. (2024). Skin cancer classification using fine-tuned transfer learning of DENSENET-121. Appl. Sci..

[26] Sarıateş M., Özbay E. (2024). A Classifier Model Using Fine-Tuned Convolutional Neural Network and Transfer Learning Approaches for Prostate Cancer Detection. Appl. Sci..

[27] Alhichri H., Alswayed A.S., Bazi Y., Ammour N., Alajlan N.A. (2021). Classification of remote sensing images using EfficientNet-B3 CNN model with attention. IEEE Access.

[28] Touvron H., Cord M., Sablayrolles A., Synnaeve G., Jégou H. Going deeper with image transformers. Proceedings of the IEEE/CVF International Conference on Computer Vision.

[29] He J., Xu H., Li S., Yu Y. (2024). Efficient SonarNet: Lightweight CNN Grafted Vision Transformer Embedding Network for Forward-Looking Sonar Image Segmentation. IEEE Trans. Geosci. Remote Sens..

[30] Mallya A., Davis D., Lazebnik S. Piggyback: Adapting a single network to multiple tasks by learning to mask weights. Proceedings of the European Conference on Computer Vision (ECCV).

[31] Bahrami M., Forouzanfar M. (2022). Sleep apnea detection from single-lead ECG: A comprehensive analysis of machine learning and deep learning algorithms. IEEE Trans. Instrum. Meas..

[32] Krishnamoorthy P., Sathiyanarayanan M., Proença H.P. (2024). A novel and secured email classification and emotion detection using hybrid deep neural network. Int. J. Cogn. Comput. Eng..

[33] Zhang H., Tang H., Sun Y., He S., Li Z. (2025). Modality-Specific Interactive Attack for Vision-Language Pre-Training Models. IEEE Trans. Inf. Forensics Secur..

[34] Li Q., Li H., Hu W., Sun S., Qin Z., Chu F. (2024). Transparent operator network: A fully interpretable network incorporating learnable wavelet operator for intelligent fault diagnosis. IEEE Trans. Ind. Inform..

[35] Diallo R., Edalo C., Awe O.O., Awe O.O., Vance E.A. (2025). Machine Learning Evaluation of Imbalanced Health Data: A Comparative Analysis of Balanced Accuracy, MCC, and F1 Score. Practical Statistical Learning and Data Science Methods.

[36] Anand A., Kadian T., Shetty M.K., Gupta A. (2022). Explainable AI decision model for ECG data of cardiac disorders. Biomed. Signal Process. Control.

[37] Kumru E., Ugurlu G., Sevindik M., Ekinci F., Güzel M.S., Acici K., Akata I. (2025). Hybrid Deep Learning Framework for High-Accuracy Classification of Morphologically Similar Puffball Species Using CNN and Transformer Architectures. Biology.

[38] Jiang Z.H., Hou Q., Yuan L., Zhou D., Shi Y., Jin X., Wang A., Feng J. (2021). All tokens matter: Token labeling for training better vision transformers. Adv. Neural Inf. Process. Syst..

[39] Khan S., Naseer M., Hayat M., Zamir S.W., Khan F.S., Shah M. (2022). Transformers in vision: A survey. ACM Comput. Surv. (CSUR).

[40] http://tiny.cc/xz3n001.

[41] http://tiny.cc/i54n001.

[42] Alzubaidi L., Zhang J., Humaidi A.J., Al-Dujaili A., Duan Y., Al-Shamma O., Santamaría J., Fadhel M.A., Amidie M.A., Farhan L. (2021). Review of deep learning: Concepts, CNN architectures, challenges, applications, future directions. J. Big Data.

[43] Wang R., Chen R., Yan H., Guo X. (2025). Lightweight concrete crack recognition model based on improved MobileNetV3. Sci. Rep..

[44] Dong H., Zheng K., Wen S., Zhang Z., Li Y., Zhu B. (2024). Lightweight Ghost Enhanced Feature Attention Network: An Efficient Intelligent Fault Diagnosis Method under Various Working Conditions. Sensors.

[45] Pranta A.S.U.K., Fardin H., Debnath J., Hossain A., Sakib A.H., Ahmed M.R., Haque R., Reza A.W., Dewan M.A.A. (2025). A Novel MaxViT Model for Accelerated and Precise Soybean Leaf and Seed Disease Identification. Computers.

[46] Tripathi A., Alkhayyat A., Bhatt A.K., Sharma M., Sheikh T.H. Modeling EfficientNet-B3 model for AI-based COVID-19 detection in chest X-rays. Proceedings of the Second International Conference on Computing and Communucation Networks.

[47] Abimannan S., El-Alfy E.S.M., Chang Y.S., Hussain S., Shukla S., Satheesh D. (2023). Ensemble multifeatured deep learning models and applications: A survey. IEEE Access.

[48] Rane N., Choudhary S.P., Rane J. (2024). Ensemble deep learning and machine learning: Applications, opportunities, challenges, and future directions. Stud. Med. Health Sci..

[49] Nespoli A., Leva S., Mussetta M., Ogliari E.G.C. (2022). A selective ensemble approach for accuracy improvement and computational load reduction in ANN-based PV power forecasting. IEEE Access.

[50] Arpit D., Wang H., Zhou Y., Xiong C. (2022). Ensemble of averages: Improving model selection and boosting performance in domain generalization. Adv. Neural Inf. Process. Syst..

[51] Fang H., Guo S., Zhang P., Zhang W., Wang X., Liu S., Du P. (2023). Scene change detection by differential aggregation network and class probability-based fusion strategy. IEEE Trans. Geosci. Remote Sens..

[52] Maulana A., Noviandy T.R., Suhendra R., Earlia N., Prakoeswa C.R., Kairupan T.S., Idroes G.M., Subianto M., Idroes R. (2024). Psoriasis severity assessment: Optimizing diagnostic models with deep learning. Narra J..

[53] Gao Y., Liu J., Li W., Hou M., Li Y., Zhao H. (2023). Augmented grad-cam++: Super-resolution saliency maps for visual interpretation of deep neural network. Electronics.

[54] Kalkan M., Guzel M.S., Ekinci F., Akcapinar Sezer E., Asuroglu T. (2024). Comparative Analysis of Deep Learning Methods on CT Images for Lung Cancer Specification. Cancers.

[55] Fantozzi P., Naldi M. (2024). The explainability of transformers: Current status and directions. Computers.

[56] Zolfaghari M., Sajedi H. (2024). Automated classification of pollen grains microscopic images using cognitive attention based on human Two Visual Streams Hypothesis. PLoS ONE.

[57] Mansourvar M., Funk J., Petersen S.D., Tavakoli S., Hoof J.B., Corcoles D.L., Pittroff S.M., Jelsbak L., Jensen N.B., Ding L. (2024). Automatic classification of fungal-fungal interactions using deep leaning models. Comput. Struct. Biotechnol. J..

[58] Sălăgean G.L., Leba M., Ionica A.C. (2025). Seeing the Unseen: Real-Time Micro-Expression Recognition with Action Units and GPT-Based Reasoning. Appl. Sci..

[59] Chabalala Y., Adam E., Ali K.A. (2023). Exploring the effect of balanced and imbalanced multi-class distribution data and sampling techniques on fruit-tree crop classification using different machine learning classifiers. Geomatics.

[60] Miller T., Michoński G., Durlik I., Kozlovska P., Biczak P. (2025). Artificial Intelligence in Aquatic Biodiversity Research: A PRISMA-Based Systematic Review. Biology.

